# Experiences in Surgical Closure of Atrial Septal Defect with Anterior Mini-Thoracotomy Approach

**DOI:** 10.15171/jcvtr.2014.008

**Published:** 2014-09-30

**Authors:** Bahador Baharestani, Shahabedin Rezaei, Farshad Jalili Shahdashti, Gholamreza Omrani, Mona Heidarali

**Affiliations:** ^1^Interventional Research Center, Rajaie Cardiovascular Medical and Research Center, Iran University of Medical Sciences, Tehran, Iran; ^2^Rajaie Cardiovascular Medical and Research Center, Iran University of Medical Sciences, Tehran, Iran; ^3^Cardiac Electrophysiology Research Center, Rajaie Cardiovascular Medical and Research Center, Iran University of Medical Sciences, Tehran, Iran

**Keywords:** Minimally Invasive, Surgical Procedures, Sternotomy, Congenital Heart Defect, Atrial Septal Defects

## Abstract

*Introduction:* Anterior mini-thoracotomy approach is a good alternative to median sternotomy in Atrial Septal Defect (ASD) repair. Our purpose is to explain the details of our technique and peresent the results.

*Methods:* Seventy five patients with ASD (52 female and 23 male) were operated with anterior mini-thoracotomy approach in our tertiary center between March 2012 and March 2014. The mean age was 14±10 ranged from 2 to 42 years. Outcomes were defined according to cardiopulmonary and aortic cross-clump time, intensive care unit stay time, morbidity, mortality, the size of incision, the amount of post-operative bleeding, need for blood transfusion and reoperation.

*Results:* Mean Cardiopulmonary bypass time was 49.62 minutes (26 to 105 minutes) and mean aortic cross clamp time was 22.29±6.77 minutes (11 to 47 minutes). The mean amount of blood transfusion was 47.49± 62.22 mm (0 to 200 cc) and the mean chest tube drainage after surgery was 80.17 ±121.06 mm (0 to 600 cc). One patient re-operated for dehiscence of ASD surgical sutures and there was no reoperation for surgical bleeding or tamponade drainage in these patients. In 74 cases the defect was secundum type, in 2 patients it was sinus venosus type and in one with associated partial Anomalous repair.

*Conclusion:* Anterior thoracotomy approach is safe and may be the surgical technique of choice for secundum ASD repair in all age groups and we can utilize this technique also for more complicated kinds of surgery for instance, sinus venosus type ASD with or without Partial Anomalous Defect.

## Introduction

Atrial Septal Defect (ASD) is a common congenital anomaly and without repair can induce irreversible sequels in patients. Per-cutaneous closure of defect with amplatzer is the technique of choice in the most frequent form of ASD (secundum type) but if this modality cannot be used because of the lack of borders in the foramen or failing the technique, invasive surgery is recommended. In the most traditional form of surgery midline sternotomy and use of total Cardiopulmonary Bypass (CBP) with ascending aorta and Superior Vena Cava (SVC) and Inferior Vena Cava (IVC) cannulation is recommended. Recently anterolateral thoracotomy techniques with peripheral cannulation of aorta , IVC and SVC is discussed by some authors, but peripheral cannulation needs one more incision and it is more complicated in very low weight children or in fat peoples and has its own risks of peripheral vessels damage. Also in these modalities the need for long and narrow cannulas and anesthesiologists help and for cervical cannulation of SVC and also the need for negative pressure pump reservoir is mandatory that would cost more. Also device closure of secundum ASDs is sometimes needed in young children; however, little is known about the safety and outcome of this procedure in infants.^1-3^

In this study, the safety and efficacy of secundum ASD closure with anterior thoracotomy approach was evaluated. Since the trends of these young groups of patients are for minimal incisions, we would choose a technique by right anterior mini-thoracotomy approach with single sub-mammillary incision and central cannulation of ascending aorta, SVC and IVC. Surgical techniques and our 2 years of experiences and results are discussed in details.

## Materials and methods

ASD repair was attempted in 75 patients through anterior mini-thoracotomy approach in our tertiary research center between March 2012 and March 2014. All patients were operated by a single cardiac surgeon. Age ranged from 2 to 42 years; mean 14.06±10.49, 52 were female (69.3%).Their weights ranged from 7 to 82 kg and their heights from 76 to 180 cm.

### Surgical technique

Single lumen endotracheal intubation was performed for all the patients. One longitudinal pad was used under right shoulder of patients with about 20 degree elevation for better exposure. The skin incision (4-8 cm length) in right anterior 4^th^ or 5^th^ intercostals space, exactly in the mid part position between right clavicle and lower part of the anterior ribs was performed with minimal rib spreading. First, we prefer to open the chest with a 2 cm incision and estimate the position of pericardium in small sized patients, heart and diaphragm and if it is in the mid-portion between diaphragm and ascending aorta, then the incision can be expanded, otherwise we can go one space up or down to achieve the best exposure to both inferior vena cava and ascending aorta. A rib retractor should be employed and the right lung should be retracted posteriorly with a wet long gaze or a gaze suitable to the size of the patients. We open Pericardium 2 cm above phrenic nerve and in the cases with very large right thymus lobe; a partial thymectomy can be utilized for better exposure, Then we can cut a piece of pericardium for subsequent closure of ASD, and the stitch sutures can be used for retraction of pericardium for better exposure. Central cannulations of aorta were performed in all except 3 and the superior vena cava and inferior vena cava were cannulated from this single incision in all patients. We put the aortic cannula in the top of incision and the two venous cannulas in the lower part of our incision; there would be no interfering with good exposure.

**Figure 1:**
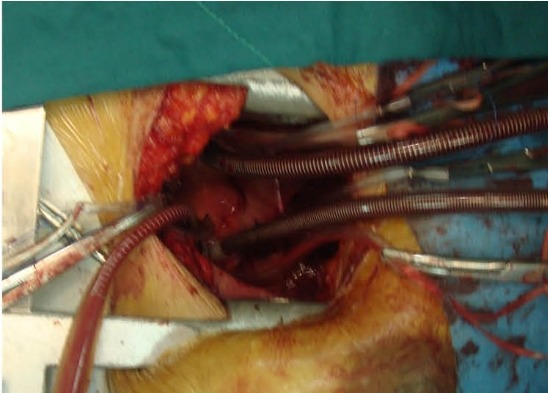
Patient cannulation

The incision is single anterior thoracotomy and we perform this kind of cannulation for all patients from 12 months (6 kg) child with a narrow femoral artery and vein to a 100 kg weighed 40 years old lady with very bad femoral exposure. After the systemic administration of the heparin, cannulation of ascending aorta with downward traction of aorta by a simple clump superior vena cava after expose it above the reflection of pericardium and right atrium near inferior vena can be done. We used to use minimally invasive instruments, although simple instruments are enough for use. We generally cannulate ascending aorta directly by 10 to 18 French Edwards Life Science Fem Flex Femoral Arterial Cannula depending on patient’s weight (Figure 1).

Superior vena cava would be surrounded with tape for ligation before we cannulate it and we surround inferior vena cava with tape after beginning of CBP and stopping ventilation. Superior vena cava and Inferior vena cava cannulate by malleable venous cannulas. After starting CBP and stopping ventilation and sending a tape around the inferior vena cava in normothermia and without the use of Co_2_, ascending aorta was cross clamped with a DeBakey type Trans-thoracic aortic clamp through the third intercostals space in the mid-axillary line. This incision could be used for later chest tube insertion but we clamped the aorta from our thoracotomy incision directly in most of the cases.

**Figure 2:**
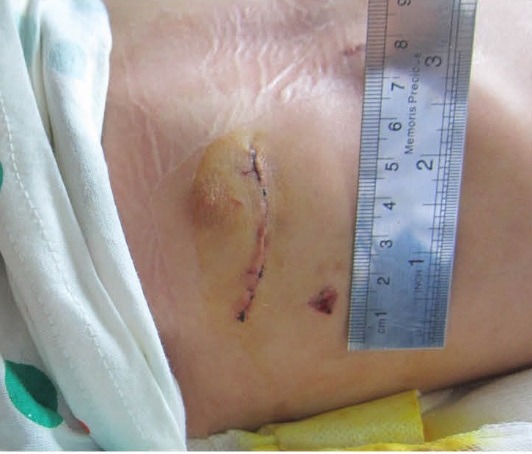
Final surgical result

Myocardial protection would be achieved with ante grade cardioplegic solution infusion through an angiocath at the ascending aorta, then right atrium opene and stay sutures are used for better exposure. After examination of both mitral and tricuspid valves we can repair the defect with pericardial patch. In first cases, we closed ASD directly but because we had a case with detachment of ASD closure in suture lines, now we almost always use a pericardial patch for the closure of ASD. After de-airing of left atrium from the last suture, atrium closure and de-airing of ascending aorta from the insertion point of cardioplegia and de-clamping of Aorta, we can close this point with a simple stitch. After discontinuation of CPB, de-cannulation, administration of protamine, hemostasis, and chest tube insertion, the incision can be closed in 4 layers’ of soft tissue and sub-coetaneous stitches for skin closure (Figure 2). We did not use rib stitches in this group patients because it results in more post-operative pain.

## Results

We had no early postoperative mortality. CPB time was 26 to 105 minutes (mean 49.62±15.71 minutes). Aortic cross-clamp time can also imply the difficulty of a cardiovascular surgery and were from 11 to 47 minutes (mean 22.29±6.77 minutes). Incision sizes were from 4 to 8 cm (mean 4.98±1.01 cm). Intensive Care Unit stay time was from 2 to 6 days (mean 2.57 ±0.79 days) and the mean amounts of blood transfusion during and after operation were 47.49±62.22 milliliters range from 0 to 200 cc and mean post-operative chest tube drainage were 80.17±121.06 ranges between 0 to 600 cc (Table 1). There was no case of reoperation for surgical bleeding or cardiac tamponade in these groups of patients. 72 ostium secundum type defects and 2 sinus venosus type ASD [(one of them was associated with Partial Anomalous Pulmonary Vein Connection (PAPVC)] were repaired and one patient was re-operated for dehiscence of ASD suture line. Further cardiac pathologies were mild tricuspid valve regurgitation in 11, moderate mitral valve regurgitation in 2 and PAPVC in one patient. Minimal residual ASD without significant shunt were seen in 4 patients with post-operative trans-thoracic echocardiography and one reoperation was the only post-operative complication that was done because primary (without patch) ASD repaired sutures were detached in 5^th^ post-operative day and because of epilepsy this patient stayed in intensive care unit for 6 days after reoperation.

**Table 1:** Patients Clinical Characteristic

	**Minimum**	**Maximum**	**Mean±SD**
CPB Time (min)	26	105	49.62±15
Aortic cross clamp time (min)	11	47	23.32±11
Blood transfusion (cc)	0	200	47.49±63
Incision size (cm)	4	8	4.49±1
ICU stay time (days)	2	6	2.57±0.79
Post-op chest tube drainage (cc)	0	600	80.17±121

## Discussion

Lancaster et al. compared thoracotomy with sternotomy in surgical closure of ASD.^4^ Izzat et al. used mini-thoracotomy approach in ASD closure.^5^ Percutaneous approach was first introduced by Bennhagen et al. in 2001.^6^ Butera et al. compared per-coetaneous closure of ASD with surgical closure and described their indication.^7^ Chang et al. compared sternotomy with thoracotomy approach in ASD closure.^8^ Mishra et al. compared mini-thoracotomy with trans-catheter approach in ASD closure.^9^ We used mini-thoracotomy approach in 76 cases in last 2 years. we approached all our ASD cases from anterior mini-thoracotomy in the last 2 years and there were no sternotomy cases for comparison. In Mishra group, lateral thoracotomy with peripheral cannulation was used and our incision is more acceptable because of aesthetics reasons and especially in ladies it can be hidden behind breast. All our patients were operated by one incision and we cannulated all cases from this incision from 12 months (6 kg) child with a narrow femoral artery and vein to a 100 kg weighed 40 years old lady with very bad femoral exposure. The only complication was a case of dehiscence of sutures that was operated by primary closure of ASD and after this complication, we preferred to use pericardial patch for all our cases for reducing tension on suture lines. CPB time is a useful operative measure in cardiac surgery and aortic cross-clamp time which can also imply the difficulty of a cardiovascular surgery, was both acceptable and a little longer than Mishra group, but we closed all Foramens with a pericardial patch in our experience that needs a little more time and our incisions were shorter which requires us to be more precise in operation (mean 4.98 cm in our group and 8 cm in Mishra group). There was no case of surgical drainage or blood or fluid induces tamponade in our experience. Our first cases complained of chest wall pain and nipple numbness but after discontinuation of rib suture usage, these complaints discontinued. Now we close chest-wall fascia in 2 layers and sub-coetaneous and skin in 2 other layers.

## Conclusion

Anterior mini-thoracotomy approach for repair of ASD brings benefits to patients and is very practical if designed correctly, even though it is a more complex surgical procedure with longer CPB and aortic cross-clamp time, it can be the preferred approach if the surgeon has had enough experiences .

In our point of view, anterior thoracotomy approach is safe and may be the surgical technique of choice for secundum ASD repair in all age groups and we can utilize this technique also for more complicated kinds of surgery for instance, sinus venosus type ASD with or without partial anomalous defect. After all, this approach neither needs additional instruments nor more expenses.

## Ethical issues

This study was approved by our local Ethics Committee.

## Competing interests

Authors declare no conflict of interest in this study.
